# A Machine Learning Approach to Identify the Importance of Novel Features for CRISPR/Cas9 Activity Prediction

**DOI:** 10.3390/biom12081123

**Published:** 2022-08-16

**Authors:** Dhvani Sandip Vora, Yugesh Verma, Durai Sundar

**Affiliations:** 1 Department of Biochemical Engineering and Biotechnology, Indian Institute of Technology Delhi, Hauz Khas, New Delhi 110016, India; 2 Yardi School of Artificial Intelligence, Indian Institute of Technology Delhi, Hauz Khas, New Delhi 110016, India

**Keywords:** CRISPR/Cas9, genome editing, machine learning, SHAP values, binding energy, off-targets

## Abstract

The reprogrammable CRISPR/Cas9 genome editing tool’s growing popularity is hindered by unwanted off-target effects. Efforts have been directed toward designing efficient guide RNAs as well as identifying potential off-target threats, yet factors that determine efficiency and off-target activity remain obscure. Based on sequence features, previous machine learning models performed poorly on new datasets, thus there is a need for the incorporation of novel features. The binding energy estimation of the gRNA-DNA hybrid as well as the Cas9-gRNA-DNA hybrid allowed generating better performing machine learning models for the prediction of Cas9 activity. The analysis of feature contribution towards the model output on a limited dataset indicated that energy features played a determining role along with the sequence features. The binding energy features proved essential for the prediction of on-target activity and off-target sites. The plateau, in the performance on unseen datasets, of current machine learning models could be overcome by incorporating novel features, such as binding energy, among others. The models are provided on GitHub (GitHub Inc., San Francisco, CA, USA).

## 1. Introduction

Clustered regularly interspersed short palindromic repeats (CRISPR) and its associated nuclease Cas9 constitute a versatile and reprogrammable genome editing mechanism that has been repurposed as a widely used tool [1,2,3]. The single guide RNA can be customised to target the DNA at any location by changing the 20 nucleotides “spacer”. This spacer is designed to complement the “protospacer” region in the DNA, at which the Cas9 nuclease would create a double-stranded break [4]. A 3-nucleotide protospacer adjacent motif (PAM) is a prerequisite for probing and cleaving the target DNA by this two-component protein–RNA system [1]. The PAM site is generally of the form of NGG (where N is any nucleotide) for the *Streptococcus pyogenes*-derived Cas9 (SpCas9) protein [5,6]. The SpCas9 is a multidomain protein consisting of (i) three recognition domains that bind to the RNA and DNA strands, (ii) two nuclease domains to cleave each of the DNA strands, (iii) a PAM interaction domain, and (iv) an arginine-rich helix which acts as a linker [7]. Although this system is a facile and flexible genome editing tool, there are two critical design problems associated with this system: (i) designing a guide RNA with good activity at the intended target region and (ii) ensuring that the selected guide does not show activity at similar unintended sites, or in other words, has low off-target activity [8,9]. The presence of the Cas9 off-target activity has hindered clinical applications of Cas9, which is a significant area of focus for CRISPR/Cas9 study.

Great strides have been taken to understand the mechanism of action and, consequently, develop design rules to aid experimentalists in optimising guides for the intended applications. The field has benefited greatly over the past decade, majorly because of the development of multiple methods to detect Cas9 off-target activity in vitro and in situ within the cell [10,11,12,13,14,15]. Off-target detection techniques have enabled the identification of empirical rules that seem to drive off-target identification and activity by allowing analyses of various off-targets generated for multiple guides under different conditions [16,17,18,19].

The availability of an experimentally derived structure and sequence of target and off-target data has allowed computational studies to understand Cas9 activity. Many prediction algorithms have been proposed to achieve each of the tasks mentioned above, qualitative algorithms and scoring schemes to rank guides by on-target efficiency and off-target predictions [20,21]. Most algorithms are based on sequence features—number and position of mismatches (PAM proximal ends are less likely to tolerate mismatches, while the distal ends report more tolerance for mismatches) [17]. Many machine learning models have been built to predict the performance of guides and the prediction of their respective off-targets based on rules depending on the system’s various sequence and structural features [17,22,23,24,25], yet there is a gap between the predictions and experimentally observed results. Popular machine learning models are based on features such as the sequence at the cut-site, the number of mismatches, experimentally validated efficiency and off-target activity of the guides. Recently, deep learning models have been reported, which are trained on large-scale datasets, and some have included novel features for validation; for example, DeepCRISPR, one of the earlier attempts at building a deep learning-based tool for prediction, introduced four epigenetic features apart from the sequence features [26]. DeepCpf1 is a convolution neural net (CNN) model, and CRISPcut is a rule-based model, both of which include chromatin accessibility as an additional feature to improve the prediction confidence [27,28]. CRISPcut and AttnToCrispr are prediction algorithms that also have included the cell-line information as features while predicting off-targets and on-target efficiency, respectively [28,29]. The addition of new and important features has, in each case, improved the model performance and confidence in the predictions. Recent studies have reported that DNA enthalpy (a proxy for the stability of the DNA duplex) and DNA-RNA duplex energy parameters play an essential role in predicting on-target efficiency and off-target activity [24,30]. This study presents two new features that prove to be important in future prediction algorithm designs: MMGBSA-based binding energy for (i) DNA and guide RNA, and (ii) Cas9 protein–nucleic acid recognition domain and the DNA-RNA hybrid.

## 2. Materials and Methods

### 2.1. Data Assembly

The data used for model training and validation were obtained from published methods of CRISPR/Cas9 off-target site prediction (CRISPcut) [28] and detection (CIRCLE-seq) [11,28] (SRA identifier SRP103697). The predictions obtained from CRISPcut, run with default parameters, for the 11 guide RNAs used in CIRCLE-seq were used to obtain a comprehensive list of potential off-target sites in the genome for the corresponding cell lines used in the CIRCLE-seq experiment. The experimentally validated off-target sites were called the positive dataset, while the predictions not validated experimentally were referred to as the negative dataset. All predictions obtained from CRISPcut were analysed for chromatin accessibility; only accessible sequences were selected since earlier studies have established the importance of this feature [31,32,33]. The data assembly and selection are summarised in Table S3. The cleavage efficiency obtained from the CIRCLE-seq dataset for all reported off-targets was normalised to fit a uniform scale. The features used for model training are detailed in Table S4.

### 2.2. Predictive Features

Multiple predictive features were calculated for each of the sequences—mismatch position, number of mismatches, mismatch in PAM, type of mismatch (transition, transversion or indel), cell line information, percentage GC for the protospacer, percentage GC in the seed region, chromosome number, DNA strand information and the two new proposed binding energy features. Two MMGBSA-based binding energy features were considered—dG(REC3:hybrid) and dG(DNA:RNA). The dG(REC3:hybrid) was calculated between the REC3 domain of SpCas9 and the 20-nucleotide DNA-RNA hybrid. The binding energy of the 20-nucleotide RNA and target DNA strands was calculated as dG(DNA:RNA). The MMGBSA calculations were carried out using the Schrödinger Maestro suite’s Prime utility after pre-processing and the restrained minimisation of the complexes [34,35].

### 2.3. MMGBSA Binding Energy Calculation

The structure used as a template was obtained from RCSB PDB (ID: 4UN3). The REC3 domain was selected (residues 447–718) along with the 20 nucleotides of the target DNA and the 20 nucleotides of the guide RNA. The PyMOL nucleic acid mutagenesis tool was used to create all target and off-target systems from the template [36]. The structures were imported in the Schrödinger Maestro suite and preprocessed, hydrogen bonds were optimised, and restrained minimisation was carried out before performing MMGBSA calculation using the Prime utility [34,37]. The energies of molecular mechanics when combined with the generalized Born and surface area continuum solvation (MMGBSA) is a popular approach to estimate the binding free energy between biomolecules. MMGBSA is an intermediate in both computational costs and accuracy, widely applied for various systems [38,39,40]. The free energy is calculated and summed over solvation energy, gas-phase energy and entropic contributions. The REC3 domain was chosen as the receptor and the DNA-RNA hybrid was used as the ligand for the dG(REC3:hybrid) feature; DNA was selected as the receptor for the dG(DNA:RNA) feature.

### 2.4. Mann–Whitney U Test

The Mann–Whitney U test, also called the Mann–Whitney–Wilcoxon test, is a non-parametric test to compare differences of a variable between two groups when the variable in question is not normally distributed. The test was performed on the dataset for both dG features, the values of which served as input for the test enabled by the Pingouin Python package (0.5.2) [41]. The common language effect size was calculated using a Python script. The output is a U statistic and *p*-value, which indicates whether the groups show stochastic equality or not. The test is also robust to outliers. The U test was used to determine if the dG values for the experimentally validated off-targets (positive) and the non-validated predictions (negative) were statistically different.

### 2.5. Machine Learning Model Implementation

Two machine learning models were implemented:

(1) A random forest regression model on a small fraction of the CIRCLE-seq dataset with the dependant variable as normalised cleavage frequencies following their normalisation;
(2) A random forest classification model on a fraction of the CIRCLE-seq and CRISPcut derived datasets with the dependant variable being whether the sequence is cleaved experimentally or not.

The regression model was to determine whether the binding energy features significantly impact the cleavage frequency of the off-target sequences. The classification model would help determine if the energy features play a role in differentiating experimentally unlikely predictions from experimentally validated off-target sequences. Since the dG values calculation was computationally intensive and time consuming, the dataset consisted of 186 positive examples and 126 negative examples. However, the sequences were collected manually to ensure sufficient diversity in cleavage frequency, the number of mismatches, and other sequence features that were previously reported as significant. The classification model was implemented to understand if the features were sufficient to differentiate between experimentally likely predictions and those that are not.

Multiple machine learning models were tested with varying parameters; the best performing models were reported. All models evaluated were implemented using the scikit-learn package in Python [42].

### 2.6. Sampling Data for Training

Initial training was performed on a 75% train set, and assessment of the model performance was measured on the 25% held-out test dataset. The best performing model architecture was selected. For analysis of feature importance, since the dataset was limited, training was carried out again with 5-fold cross validation to ensure that the unbalanced dataset was not a limiting factor for model performance. The 5-fold cross-validation was repeated to ensure the absence of bias for both models.

### 2.7. Assessing Model Performance

The regression model’s performance was evaluated by comparing the mean squared error (MSE), mean absolute error (MAE) and the R-squared values, and the better performing model was selected for feature importance determination and feature ranking. The MAE and MSE measure the difference between the model predictions and actual observations; hence, the ideal score is 0. The R-squared value is a correlation coefficient measuring a linear correlation between two continuous variables. The variance weighted measure is an explanation of the variance in the model output, the best score being 1.

The classification model was assessed using its confusion matrix:

$$M=\left[\begin{array}{cc} TP & FN\\ FP & TN \end{array}\right]$$

where *TP* stands for true positive, *FP* for false positive, *FN* for false negative and *TN* for true negative. The accuracy of a model is defined as

$$\mathrm{Accuracy}=\frac{TP+TN}{TP+TN+FP+FN}$$

The recall is the measure of how many actual positives the model can capture, while the precision is how many of the predicted positives are correct. The precision–recall curve, a standard evaluation criterion for a classification model, is based on the following definitions:

$$\mathrm{Recall}=\frac{TP}{TP+FN}$$

$$\mathrm{Precision}=\frac{TP}{TP+FP}$$

The F1 score, or F-measure, is the harmonic mean of the precision and recall, conveying a balance between the two. It is defined as

$$F1\;\mathrm{score}=\frac{2*Recall*Precision}{Recall+Precision}$$

### 2.8. Identifying Feature Importance

Interpreting the features that impact a machine learning model’s outcome is important for enabling the predictions’ validation. In the regression and classification models used, the feature set is small, and so is the dataset; hence, each feature’s influence must be understood. Hence, Shapley additive explanations (SHAP) values were implemented using the shap library in Python [43]; the TreeExplainer utility was used to analyse the random forest regressor output and to describe the model output of the random forest classifier [44]. The shap method employs an explanatory model with feature weights to explain relative feature importance and is adapted from game theory. It is to be noted that shap values do not indicate causality.

## 3. Results

### 3.1. Data Assembly and Processing

The guide RNAs and their respective off-targets were obtained from the CIRCLE-seq data [11]. The data obtained from the prediction algorithm CRISPcut were checked for the number of sites predicted for each guide RNA input [28]. The number of sites predicted hold little correlation with the experimental sites (Figure S1a). However, when the chromatin accessible sites were selected and compared, a sufficient correlation was obtained between the number of sites predicted and the number of sites confirmed experimentally (Figure S1b). Moreover, since chromatin accessibility has been shown in earlier studies to be an important feature, sequences selected for the model were only from the accessible sequences’ subset [31,45].

The sequences selected from the CIRCLE-seq (positive dataset) and CRISPcut predictions, but not found in the experimentally validated datasets (negative dataset), were selected manually to ensure that the other features, such as the number of mismatches, cleavage frequencies and cell lines, were sufficiently represented. The features included for the model prediction were calculated using Python scripts, except the binding energy features, which were calculated using the method described. The resulting dataset had 40 features and 312 data points.

To determine if the features were correlated with each other, correlation analysis was carried out, and the results are shown in Figure S2. No significant correlation between the features was observed. The correlation islands observed were between the cell lines that were one-hot encoded and are hence mutually exclusive. A high correlation was expected for the total mismatches and protospacer mismatches (referred to as number of mismatches, #mm); the same can be stated for total PAM mismatches and types of PAM mismatches—transversion or transition type. Hence, the features selected were unique and not redundant.

### 3.2. Statistical Analysis of the Binding Energy Features

To determine if the values of the binding energies, by themselves, could be used to differentiate between the positive and negative datasets, the Mann–Whitney U test was carried out to compare the values between the two sets (Supplementary Table S1). The Mann–Whitney U test is a non-parametric test to check if a feature’s values are larger for one of the two populations being compared; it is the non-parametric equivalent of the unpaired *t* test.

The values of the two binding energy features were compared for the positive and negative datasets, where the H_0_ hypothesis was that the values for the two groups are equal. Hence, the H_0_ hypothesis’s rejection indicated that the difference between randomly selected values of the features from both populations is big enough to be statistically significant (Table S1). The rank–biserial correlation coefficient indicated the difference between total amount of favourable and unfavourable evidence. The common language effect size is the probability that a random value from Group 1 is greater than a random value from Group 2.

The Mann–Whitney U (MWU) test indicated that the values of the two binding energy features—dG(REC3:hybrid) and dG(DNA:RNA)—have differing values for the positive and negative datasets (Table S1). Moreover, it is evident from the MWU test that a random value from the negative dataset is likely to be higher than a random value from the positive dataset. However, since the effect size values are low, the features cannot solely be used as a distinguishing factor for the negative and positive datasets. The difference in population means the calculation was not enough to reliably call these features distinguishing.

### 3.3. Regression Model Selection and Performance Assessment

Linear, quadratic, cubic, multi-layer perceptrons and random forest regressors were implemented with varying parameters and random states to determine the best performing model. The dependent variable was the cleavage frequency for the off-target sequences obtained from the CIRCLE-seq dataset. The performance measured in the *R*-squared value, mean absolute error, mean squared error and variance-weighted measure is summarised in Table 1. The random forest regressor was chosen based on its superior performance on the dataset, compared to the other models tested. The random forest algorithm is known for its ability to predict well on tabular data, as is the case here. The perceptron was also tested for multiple nodes in one and two hidden layers trained till convergence; however, it failed to outperform the random forest regressor.

The best performing regression model, the random forest regressor, was initialized on various random states and number of trees (as shown in Figure S3). The model with the maximum R-squared and minimum mean absolute error (MAE) was selected for further analysis, following which 5-fold cross-validation was performed. The resulting mean squared error (MSE) remained at 0.05, standard deviation (STD) was 0.01, and the R^2^ score was 0.92, indicating that the chosen model was robust.

### 3.4. Explaining Feature Importance for the Random Forest Regressor

**Figure 1:**
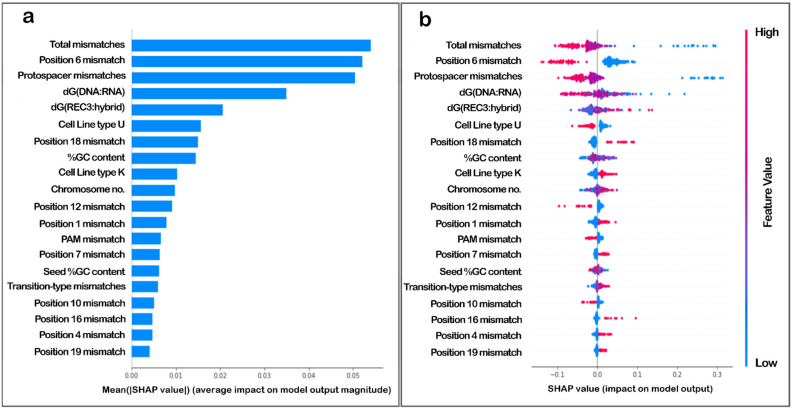
SHAP variable importance plots. (**a**) The plot arranges features in decreasing order of magnitude of impact on model output. (**b**) The features are listed in decreasing order of importance, the dots are coloured according to value (in a gradient from high to low, as red to blue) and the impact for each instance is plotted horizontally. The spread indicates impact on model output, and the colour indicates feature value for that output.

The importance and magnitude of the impact of the features on the model output were explored in detail since the aim of the study was to establish the importance of the two features proposed, namely the energy of binding of the REC3 domain of Cas9 to the 20 nucleotide hybrid of the target DNA and guide RNA-dG(REC3:hybrid), and the binding energy of the 20 nucleotide DNA to the guide RNA strand-dG(DNA:RNA). The variable importance plot (Figure 1a) generated by implementing SHAP [43,44,46] lists the most important features in descending order. The ones on top contributed the most to the model output and hence, have high predictive capability.

The SHAP values also help determine the relationship of the features to the output. The SHAP variable importance plot (Figure 1b) ranked variables in descending order of importance, and the horizontal spread indicated the effect of the value and the corresponding higher or lower prediction. Each dot is a value for an instance in the data, and the colour indicates a higher or lower value for that instance. While distance (total mismatches in the sequence) and #mm (mismatches in the protospacer region) were redundant features and showed a similar impact on output, Figure 1 shows that the low binding energy of the DNA-RNA hybrid, dG(DNA:RNA), had a high impact on model output; while the binding energy of the Cas9 REC3 domain to the DNA-RNA hybrid, dG(REC3:hybrid) was negatively correlated with the model output. Figure 1 also indicates that the presence of mismatch at the 6th position played an important role in determining the model output.

**Figure 2:**
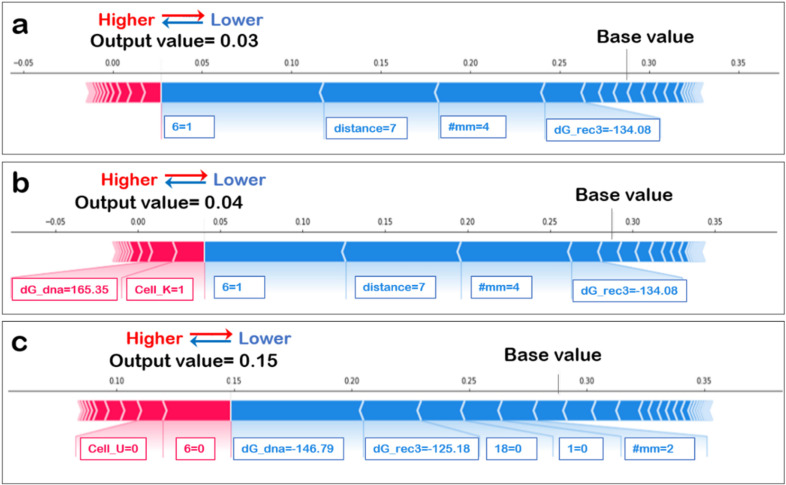
SHAP variable importance plot. The SHAP variable impact on outcome for singular datapoints are shown. Examples shown are explainer plots for dataset indices (**a**) 0, (**b**) 1 and (**c**) 2. The base value labelled in the figure in influenced by varying degrees by the features shown in the diagrams and the output value (shown in bold) was obtained. The features SHAP values are written alongside the features if it causes an increase in base value it is shown in red otherwise in blue.

The SHAP variable importance plot (Figure 2) takes three values: a base value, SHAP values, and the matrix of feature values. The base value was the average or expected model output, and the SHAP value of a feature and the value of the feature at that instance determined in which direction the features “push” the model output. The output value highlighted is the model output for this instance. The features in red direct the output higher, while those in blue push the predictions lower. The SHAP plot for three instances are shown; since each feature plays a different role for each instance, it is essential to consider the local as well as global relevance of the feature.

**Figure 3:**
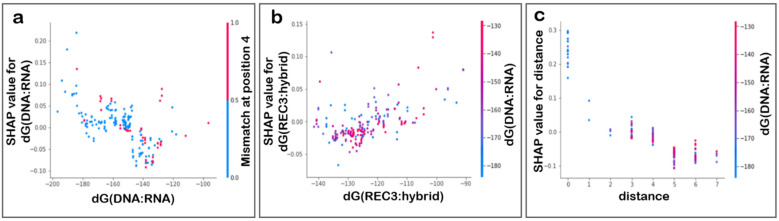
SHAP feature dependence plot. The plots show dependence between (**a**) dG(DNA:RNA) and a mismatch at position 4, (**b**) dG(REC3:hybrid) and dG(DNA:RNA) and (**c**) distance and dG(DNA:RNA). The vertical axis marks the SHAP values for the chosen feature, while the horizontal axis shows spread of the values of the feature. The reference feature was selected by the algorithms automatically and was used to colour the dots that indicate value of the primary feature for an instance. No clear trend can be observed in (**a**,**b**). In (**c**), vertical clusters at individual values indicate a correlation with dG(DNA:RNA) values, and the plot also shows a negative correlation of the values of the distance with the output variable.

The SHAP dependence plot (Figure 3) describes partial dependence between a feature selected, and the reference feature was chosen automatically by the script with which the chosen feature interacts the most. The dots mark each instance of the chosen variable, and the colour of the dots indicate the value of the reference feature for that instance. In Figure 3a,b, there is no clear trend between the two features; however, in Figure 3a the absence of a mismatch at position 4 and the lower values of dG(DNA:RNA) have a higher impact on the model output. Figure 3b shows that the partial dependence between the two features is not significant and no trend can be observed. The spread of the plot indicates the relationship between the two features. As in Figure 3c, the vertical dispersion at a particular value shows the interaction effect between the two features. Moreover, an approximately negative correlation exists between the variables, and a smaller Hamming distance (total mismatches in the off-target) would have more influence on the model output; it also corresponds with lower values of dG(DNA:RNA).

### 3.5. Classifier Model Selection and Performance Assessment

The classifier models were built to study the contribution of the binding energy features to machine learning models that can distinguish between positive (sequences that are off-target sites in experiments) and negative datasets (sequences predicted to be off-targets but were not found in experiments). Various classification models were trained on the dataset, optimised for each type of model (the best performing model’s accuracy summarised in Table S2). Since the random forest classifier performed well on the 25–75 test-train split, the model was evaluated after 5-fold cross validation. The classifier yielded good accuracy and was implemented for further analysis. The model metrics for the random forest classifier model are summarised in Table 2.

**Figure 4:**
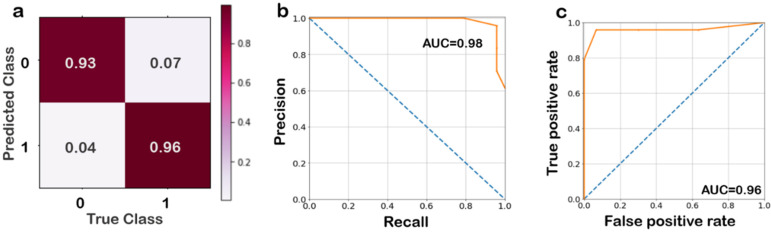
(**a**) Confusion matrix for the random forest classifier, vertical axis is for predicted labels and the horizontal axis states the true labels. The values are ratios of the number of instances predicted to the total instances in the class. (**b**) Precision–recall curve, shown in orange which has an area under the curve of 0.98 for the whole dataset, (**c**) receiver operating characteristic (ROC) also shown in orange for the test dataset, which plots the true positive rate against the false positive rate. The area under the curve (AUC) is 0.96. The dashed blue line across the diagonal shows 50% accuracy.

The performance of the random forest classifier was tested using various parameters as shown in Figure 4. The model predicted the correct classes for each label reliably. The precision–recall curve and receiver operating characteristic (ROC) cover over 95% area under the curve, indicating a robust classification model. The next best performing model (support vector machine classifier) did not perform better, even on 5-fold cross validation, and hence was not evaluated further. Since the study aimed not to build an off-target determination model, but rather discern the importance of energy features, more complex models were not tested.

### 3.6. Explaining Feature Importance for the Classifier

**Figure 5:**
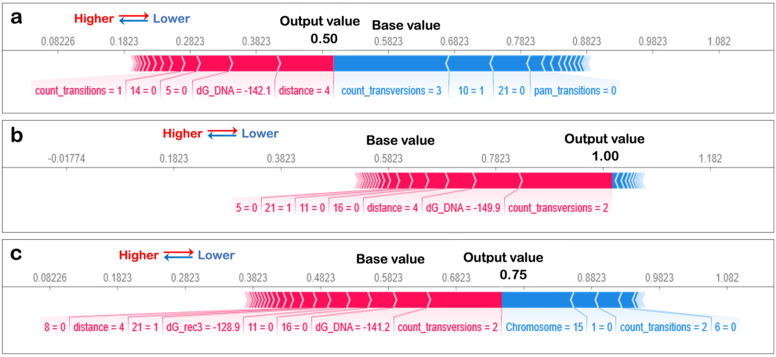
SHAP value plots for singular datapoints. Examples shown are for dataset indices (**a**) 10, (**b**) 17 and (**c**) 21, and are chosen randomly. The base value shown increases by features shown in red and decreases because of features shown in blue. Each feature impacts the value in magnitude indicated by SHAP values labelled alongside for each instance.

**Figure 6:**
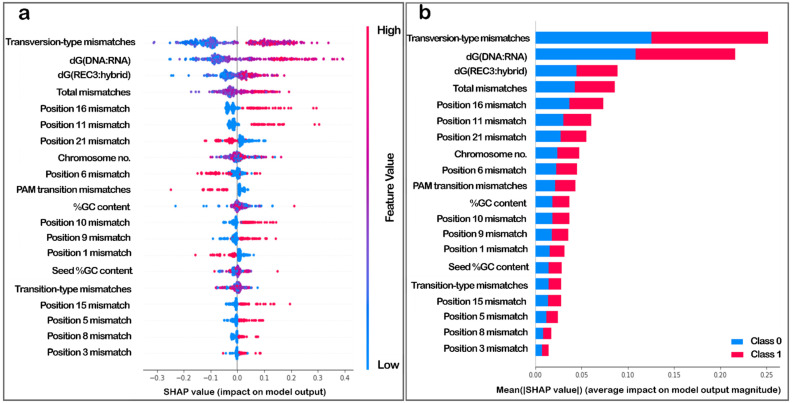
(**a**) SHAP value plot indicating global impact n model output. Each dot is an instance for a datapoint, the colour represents if the value for that instance is low (blue) or high (red). The spread indicates the magnitude of impact on the model output. (**b**) SHAP summary plot shows the impact of the features on each model output, negative class shown in blue and positive class shown in red, as stacked bars, in decreasing order of impact on output.

The importance of the features in a well-performing classification model that can learn the difference between the positive and negative datasets will determine if the binding energy features play a significant role in determining the model output. The SHAP value plots for each instance are not shown for lack of space, but three examples are shown in Figure 5. The base value, determined as the average from the training dataset, is influenced by the features listed in order of magnitude of impact. Features in blue lower the output, while features in red increase the output. In all instances, energy features play an important role. However, since feature importance for each datapoint varies, it is important to see each feature’s global impact, which is shown in Figure 6.

This SHAP value plot ranks the features in decreasing order of importance, while the spread across the horizontal determines the impact on the model for higher values (in red) and lower values (in blue). As is shown in Figure 6, the energy features are ranked high. Lower values of both binding energies are characteristic of the positive dataset. Hence, lower values of the binding energy tend to result in a positive impact on the model output; here, it is the classification in the positive dataset.

## 4. Discussion

The accurate prediction of CRISPR/Cas9 activity is crucial to not only designing experiments for various applications but also understanding the mechanism of Cas9 activity in vivo. Computational methods for predicting activity, off-targets and guide design have advanced significantly in recent times, yet there remains room for improvement regarding precision and accuracy. Prediction models would also benefit from improved and more sensitive Cas9 off-target detection methods to better distinguish between sequences likely to be acted upon by Cas9 (here, the positive dataset). This study reported that the incorporation of novel features allows for creating reliable prediction models. Moreover, the identification of novel features also sheds light on the factors influencing Cas9 activity in vivo.

The two major binding events responsible for Cas9 activity are (1) the binding of the Cas9 protein to the guide RNA, allowing DNA interrogation for complementary sequences, (2) followed by binding to the complementary sequence, which allows nuclease activation and a subsequent DNA double-stranded break [47]. Significantly accelerated by the availability of X-ray and cryo-EM structures, computational methods, such as QM/MM and molecular dynamics (MD), have elucidated the pre-catalytic and catalytic structures of Cas9 [48,49]. Enhanced MD simulations have shed light on the concerted mechanisms of HNH and RuvC domain activities [50,51,52]. The HNH domain via an Mg^2+^ ion cuts the target strand, while the RuvC domain houses two metal ions coordinated by conserved residues, which mediate a break in the non-target strand [52]. The varying tolerance of the mismatches across the guide-target heteroduplex has also been investigated [18,53,54]. The REC3 domain is known to interact with the guide RNA-target DNA complex, investigate the complementarity between the two, and tolerate mismatches [55,56]. Mismatches were seen to be tolerated towards the centre of the guide–target hybrid [53]. In contrast, mismatches towards the end of the hybrid induced an extended opening of the heteroduplex and leading to a conformational lock with the “L2” loop region [54]. Hence, the interactions of the guide RNA with target DNA and the heteroduplex with the REC3 domain of Cas9 protein have been shown to play a decisive role in nuclease activation, leading to Cas9 activity. The introduction of mismatches alters the interactions, leading to altered Cas9 activity. Understanding the factors that govern the RNA:DNA interactions is critical to elucidating biological function that it is involved in [57,58,59,60]. Hence, to quantify the interactions, DNA-RNA hybrid binding energy and Cas9-hybrid binding energy were estimated and analysed. The scores were then included as features alongside sequence features, and machine learning models were built for Cas9 activity prediction. Well-performing models were selected to analyse the importance of the new energy-based features, if any.

The random forest algorithm outperformed the others tested on both classification and regression tasks. The improved performance could be attributed to the limited number of features on each split. When compared to individual decision trees, which have a higher bias, random forests tend to perform better because of the variance reduction due to bagging. The features used, as the results describe, have minimum redundancy. The energy features prove vital in driving model output in both regression and classification tasks. This feature importance was also observed in the second-best performing classification model: a support vector-based machine classifier (a second regressor was not evaluated due to the performance being subpar, not reliable enough to study feature importance). The importance of the number of mismatches in the seed region has already been established in multiple studies [61,62]. Interestingly, a higher number of transversions was shown not to be tolerated in the experimental dataset, indicating a preference in the sequences (Figure 6a). However, a bigger dataset is required to be tested to establish this. The “distance” feature’s trend may also be inferred intuitively since lower values of total mismatches are likely to be observed in the positive dataset. The energy features’ contribution was novel and ranked high consistently in multiple results, enough to be considered important. The performance of the reported random forest classifier was also compared against existing methods for off-target prediction and was found to perform better (Figure S4).

## 5. Conclusions

In this study, the binding energy of the Cas9 REC3 domain and the 20-nucleotide DNA-RNA hybrid, and the binding energy of the 20 nucleotides of target DNA to guide RNA were novel features and proposed to be important for Cas9 activity. In the regression model, which predicts Cas9 cleavage frequency, and the classification model, which predicts Cas9 activity, both these features were shown to be important in driving model output. The same importance of the features was observed in the classification model, which can reliably distinguish between experimentally likely and unlikely off-target sequences. The other features used in the model were standard features used in most studies: the number and position of mismatches and type of mismatch, among others. The binding energy features were not redundant and did not show correlation with the other features, and hence they can be implemented in future algorithms for improved off-target prediction and guide-RNA design algorithms.

## Figures and Tables

**Table 1:** Summary of model performances. All values shown are for the test dataset.

Metrics				Regressor				
	Linear	Quadratic	Cubic	Decision Tree	SVR	MLP	XGBoost	Random Forest
Mean Absolute Error	0.19	1.23	0.51	0.21	0.19	0.19	0.17	0.06
Mean Squared Error	0.07	3.49	0.54	0.09	0.08	0.07	0.06	0.01
Root MSE	0.26	1.87	0.73	0.29	0.28	0.26	0.24	0.08
R-squared value	0.37	−32.26	−4.15	0.18	0.27	0.42	0.47	0.94
Variance weighted	0.38	−31.82	−4.13	0.24	0.27	0.47	0.55	0.94

The various model metrics listed in the first column are given for the regression models tested. For the random forest regressor, the metrics are comparatively much better than the other three. It was selected for feature importance analysis. SVR stands for support vector regressor. MSE stands for mean squared error. The values reported for each regressor is after the optimisation of individual models.

**Table 2:** Model performance of the random forest classifier, measured on test dataset.

Model Metrics	Score on Test Data	Overall Score
**Accuracy**	0.86	0.97
**Precision**	0.88	0.98
**Recall**	0.94	0.96
**F1 score**	0.91	0.97

The accuracy, precision, recall and F1 scores are calculated as mentioned in the Methods section. The accuracy reported is after 5-fold cross validation. The overall score is for combined test and train datasets.

## Data Availability

All input files and python scripts used for the data generation and analysis are available on GitHub (https://github.com/TeamSundar/crispr-cas9-dG-study).

## Supplementary Materials

The following supporting information [23,24,28,63,64,65] can be downloaded at: https://www.mdpi.com/article/10.3390/biom12081123/s1, Figure S1: Features of the predicted dataset. On the vertical axis is the number of predicted sequences and on the horizontal axis is the number of experimentally validated sequences. (a) The number of CRISPcut predictions vs. CIRCLE-seq off-targets which shows poor correlation as can be determined by the low R^2^ value of 0.22. Each dot represents a unique target sequence for which the number of experimentally validated off-targets, plotted on the *X*-axis are compared against the number of predicted off-targets using the CRISPcut tool, as plotted on the *Y*-axis (b) Shows only the number of accessible predictions plotted against number of CIRCLE-seq off-target sites for a guide, high correlation denoted by an R^2^ value of 0.84 can be observed here; Figure S2: Correlation plot of the features used for model training. The dark blue diagonal indicates self-correlation. There is a poor correlation between most feature pairs, but a few high correlation islands in dark blue and yellow colour can be seen. Since cell lines are mutually exclusive, the correlation between the cell lines will be negative. The dark blue islands are between PAM mismatches, PAM transitions and PAM mismatch positions, which can be expected; Figure S3: The mean absolute error (MAE) multiplied by 10, and R^2^ value plotted for each model tested, various models were tested with increasing n_estimators and random states. The dashed grey line marks the maximum R2 and minimum error instance, which corresponds to n_estimators of 18 and a random state of 6; Figure S4: The best-performing random forest classifier was compared with the existing off-target prediction models for predicting the off-targets of a randomly selected EMX1 locus. The precision was calculated against experimentally validated sequences obtained from CIRCLE-seq. The off-targets were obtained from the CRISPOR [1,2], CRISTA [3], Elevation [4], ge-CRISPR [5] and CRISPcut [6] webservers (accessed on 13 June 2021); Table S1: Results of the two sample Mann–Whitney U test; Table S2: Random forest classification model performance summary; Table S3: Details of negative dataset; Table S4: Complete set of features used in the model learning process.
