# Supplementary material for: A Machine Learning Approach to Identify the Importance of Novel Features for CRISPR/Cas9 Activity Prediction
Source: Biomolecules. 2022 Aug 16;12(8):1123. doi: 10.3390/biom12081123 (PMC9405635; doi:10.3390/biom12081123)
Supplement: Supplementary file 1 [file biomolecules-12-01123-s001.zip › biomolecules-1839867-supplementary.pdf]

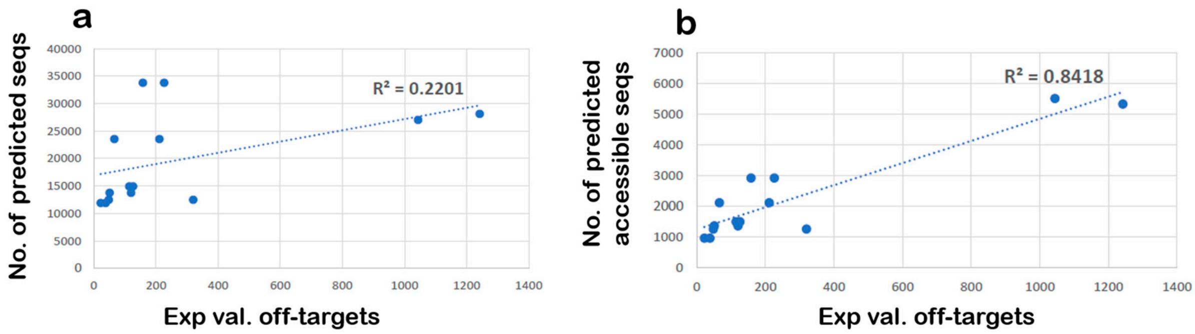

Supplementary Figure S1: Features of the predicted dataset. On the vertical axis is the number of predicted sequences and on the horizontal axis is the number of experimentally validated sequences. (a) The number of CRISPcut predictions vs. CIRCLE-seq off-targets which shows poor correlation as can be determined by the low R2 value of 0.22. Each dot represents a unique target sequence for which the number of experimentally validated off-targets, plotted on the X-axis are compared against the number of predicted off-targets using the CRISPcut tool, as plotted on the Y-axis (b) Shows only the number of accessible predictions plotted against number of CIRCLE-seq off-target sites for a guide, high correlation denoted by an R^2 value of 0.84 can be observed here.

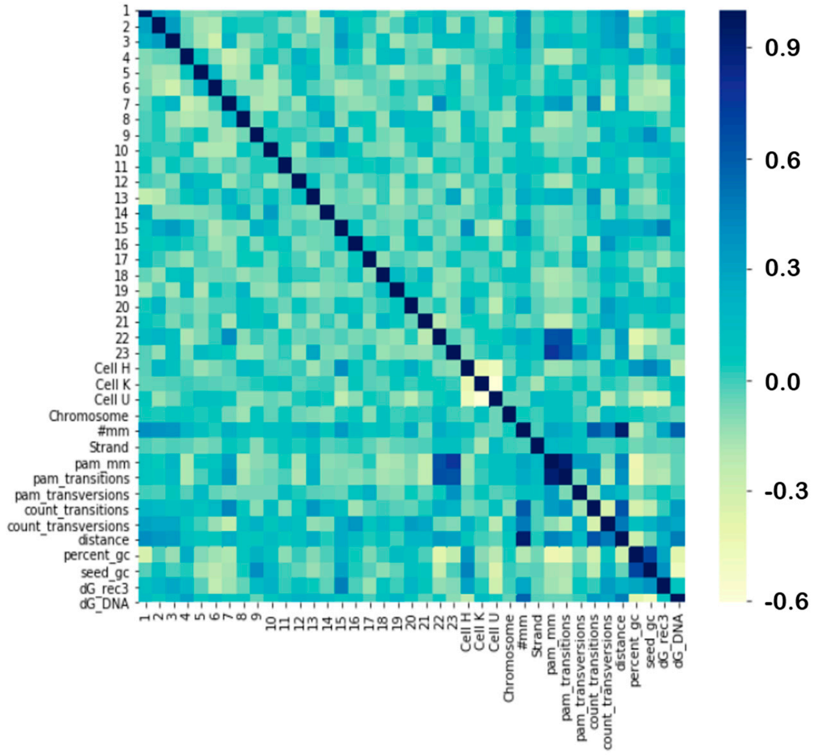

Supplementary Figure S2: Correlation plot of the features used for model training. The dark blue diagonal indicates self-correlation. There is a poor correlation between most feature pairs, but a few high correlation islands in dark blue and yellow colour can be seen. Since cell lines are mutually exclusive, the correlation between the cell lines will be negative. The dark blue islands are between PAM mismatches, PAM transitions and PAM mismatch positions, which can be expected.

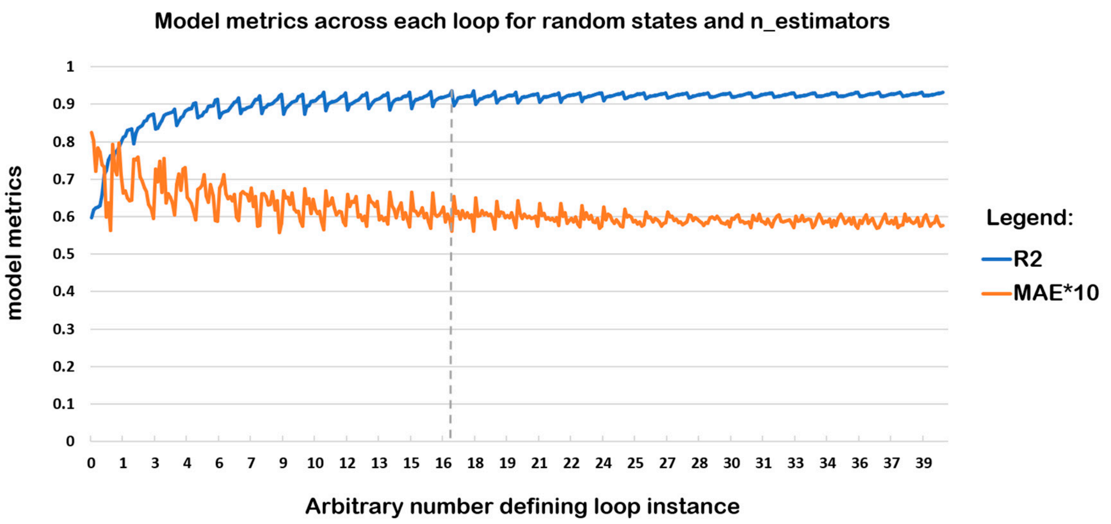

Supplementary Figure S3: The Mean Absolute Error(MAE) multiplied by 10, and R^2 value plotted for each model tested, various models were tested with increasing n_estimators and random states. The dashed grey line marks the maximum R^2 and minimum error instance which corresponds to n_estimators of 18 and a random state of 6.

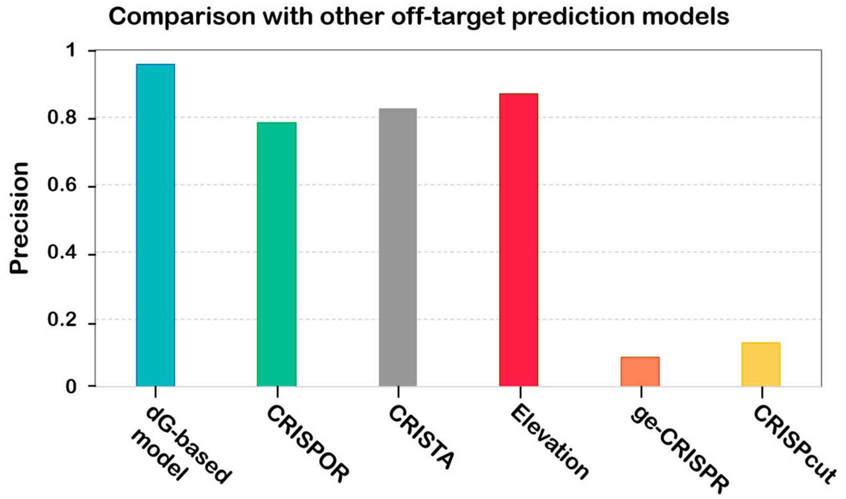

Supplementary Figure S4: The best-performing random forest classifier was compared with the existing off-target prediction models for predicting the off-targets of a randomly selected EMX1 locus. The precision was calculated against experimentally validated sequences obtained from CIRCLE-seq. The off-targets were obtained from the CRISPOR [1, 2], CRISTA [3], Elevation [4], ge-CRISPR [5] and CRISPcut [6] webservers (accessed on 13th June, 2021).

Supplementary Table S1: Results of the two sample Mann-Whitney U test.

Characteristic	dG (REC3: hybrid)	dG (DNA: RNA)
Difference in population medians	13.81	26.92
U-value	18328.00	19515.00
H_0 hypothesis	Rejected	Rejected

P-value	1.41e-17	1.01e-23
Rank-biserial correlation	-0.56	-0.67
Common language effect size	0.22	0.17

The "H_0 hypothesis" is that the two groups (positive and negative datasets) have equal dG values. The U-value, result of the Mann-Whitney test, is high indicating confidence in rejecting the H_0 hypothesis. Rejected H_0 hypothesis (as shown in the table) indicates the differences in random values selected from the two groups is statistically significant. "P-value" is less than 0.01 indicating less error and hence, confidence in the test results. The "rank-biserial correlation" is the difference in favourable and unfavourable evidences. It indicates that sufficient differences between the values of the two groups exist. The "common language effect size" values also represent that values from the negative dataset is greater than random values from the positive dataset.

Supplementary Table S2: Random Forest classification model performance summary.

Model metrics	Score on test data	Overall score
Accuracy	0.86	0.97
Precision	0.88	0.98
Recall	0.94	0.96
F1 score	0.91	0.97

The accuracy, precision, recall and F1 scores are calculated as mentioned in the Methods section. The accuracy reported is after 5-fold cross validation. The overall score is for combined test and train datasets.

Supplementary Table S3: Details of negative dataset.

Description	Number of sequences
CRISPcut predictions	199440
Accessible sites	23830
Unique sites, duplications removed	14354
Experimentally validated sequences removed, negative dataset	13802
Structures generated for dG calculations	126

The dataset predicted for the reference guides selected from the CIRCLE-seq dataset. The potential negative dataset is large, however, only a few sequences were analysed since the estimation of the energy features was time and resource intensive.

Supplementary Table S4: Complete set of features used in the model learning process.

Feature	Remarks
Mismatch at position 1	
Mismatch at position 2	

Mismatch at position 3	Mismatches were encoded in a binary form- presence (1) or absence (0) at a position indicated by a number denoting the position from the PAM distal end
Mismatch at position 4	
Mismatch at position 5	
Mismatch at position 6	
Mismatch at position 7	
Mismatch at position 8	
Mismatch at position 9	
Mismatch at position 10	
Mismatch at position 11	
Mismatch at position 12	
Mismatch at position 13	
Mismatch at position 14	
Mismatch at position 15	
Mismatch at position 16	
Mismatch at position 17	
Mismatch at position 18	
Mismatch at position 19	
Mismatch at position 20	
Mismatch at position 21	The NGG PAM was considered where N can be any nucleotide and hence no mismatch was considered, while the defaults at position 22 and 23 were 'G'
Mismatch at position 22	
Mismatch at position 23	
Number of transitions in protospacer	The type of mismatch was counted for both the protospacer and the PAM, number of indels in PAM was found to be zero and hence dropped in the later models
Number of transitions in PAM	
Number of transversions in protospacer	
Number of transversion in PAM	
Number of indels in protospacer	
Number of indels in PAM	
Chromosome number	This information was obtained from CIRCLE-seq for the positive dataset and CRISPcut for the negative dataset
Strand	

Number of mismatches in PAM	Total mismatches were counted in the PAM and protospacer regions
Number of mismatches in protospacer	
Hamming distance between the off-target and target sequences	Total distance between the two sequences was calculated, it is the sum of all mismatches and indels
Percentage GC of the protospacer	-
Percentage GC of the seed region	Seed region is considered as positions 10-20
Cell line	One-hot encoding for the three cell lines reported in CIRCLE-seq was done: HEK293, U2OS and K562
dG(REC3:hybrid)	Calculated using Schrödinger's Prime MMGBSA calculation utility
dG(DNA:RNA)	

References:

1. Haeussler, M.; Schönig, K.; Eckert, H.; Eschstruth, A.; Mianné, J.; Renaud, J.-B.; Schneider-Maunoury, S.; Shkumatava, A.; Teboul, L.; Kent, J.; et al. Evaluation of off-target and on-target scoring algorithms and integration into the guide RNA selection tool CRISPOR. *Genome Biol.* 2016, *17*, 148, doi:10.1186/s13059-016-1012-2.
2. Concordet, J.-P.; Haeussler, M. CRISPOR: intuitive guide selection for CRISPR/Cas9 genome editing experiments and screens. Nucleic Acids Res. 2018, 46, W242–W245, https://doi.org/10.1093/nar/gky354.
3. Abadi, S.; Yan, W.X.; Amar, D.; Mayrose, I. A machine learning approach for predicting crispr-cas9 cleavage efficiencies and patterns underlying its mechanism of action. *PLoS Comp. Biol.* 2017, *13*, e1005807.
4. Listgarten, J.; Weinstein, M.; Kleinstiver, B.P.; Sousa, A.A.; Joung, J.K.; Crawford, J.; Gao, K.; Hoang, L.; Elibol, M.; Doench, J.G. Prediction of off-target activities for the end-to-end design of crispr guide rnas. *Nat. Biomed. Eng.* 2018, *2*, 38–47.
5. Kaur, K.; Gupta, A.; Rajput, A.; Kumar, M. ge-CRISPR - An integrated pipeline for the prediction and analysis of sgRNAs genome editing efficiency for CRISPR/Cas system. Sci. Rep. 2016, 6, 30870, https://doi.org/10.1038/srep30870.
6. Dhanjal, J.K.; Radhakrishnan, N.; Sundar, D. Crispcut: A novel tool for designing optimal sgrnas for crispr/cas9 based experiments in human cells. *Genomics* 2019, *111*, 560–566.
